# Comparison of whole genome amplification and nested-PCR methods for preimplantation genetic diagnosis for BRCA1 gene mutation on unfertilized oocytes–a pilot study

**DOI:** 10.1186/1897-4287-11-10

**Published:** 2013-08-13

**Authors:** Danuta Michalska, Kinga Jaguszewska, Joanna Liss, Kamila Kitowska, Agata Mirecka, Krzysztof Łukaszuk

**Affiliations:** 1 INVICTA Fertility and Reproductive Center, Rajska 10, Gdansk, 80-850, Poland; 2 Faculty of Biology, University of Gdansk, Gdansk, Poland; 3 Department of Obstetrics, Gynecology and Endocrinology, University of Varmia and Masuria, Olsztyn, Poland; 4 Department of Obstetrics and Gynecology Nursing, Medical University of Gdansk, Gdansk, Poland

## Abstract

**Background:**

Preimplantation genetic diagnosis (PGD) remains nowadays a valid alternative for couples at high-risk of having a child with a genetic disease and for women older than 37–40 years with the high risk of chromosomal aneuploidies in the embryos. However the use of PGD for high penetrance recessive, dominant and X-liked disorders occurring in early life is documented, debate exists regarding its appropriateness in lower penetrance and late-onset cancer susceptibility syndromes. The data regarding the efficacy of different molecular techniques used in PGD are still lacking. We therefore sought to assess the different molecular techniques used in PGD for detecting three most frequent BRCA1 gene mutations: 5382insC, 185delAG and C61G.

**Methods:**

Anonymous donors of the oocytes and control healthy blood samples were extracted and analyzed in the Fertility and Reproductive Center Invicta in Gdansk. Preimplantation genetic diagnosis for the most frequent mutations: 185delAG, 5382insC, C61G in BRCA 1 gene was carried out on single, unfertilized oocytes, in metaphase of second meiotic division, not qualified to IVF. Positive mutation controls were represented by cell lines from the Coriell Institute for Medical Research: GM14090 (185delAG), GM14097 (C61G), GM13715 (5382insC).

**Results:**

Repeatability of the results acquired from the WGA analysis for the mutation 5382insC was 38%. The repeatability of the nested-PCR analysis in the second round of the amplification was labile for the mutation 5382insC and 185delAG and was ranged from 47% to 57%. However, the repeatability for the mutation C61G was 100%.

**Conclusions:**

Our results suggest that the nested-PCR technique remains more sensitive and specific method as compared to WGA. WGA performed on the single cells did not reflect expected results. The repeatability of the WGA methodology remains questionable, and any analysis attempt does not guarantee reliable results.

Further evaluation is strongly needed to propose the most accurate molecular technique used in PGD for detecting three most frequent BRCA1 gene mutations: 5382insC, 185delAG and C61G.

## Introduction

Preimplantation genetic diagnosis (PGD) remains nowadays a valid alternative for couples at high-risk of having a child with monogenetic diseases, i.e. cystic fibrosis, β-talasemia, Huntington’s disease, myotonic dystrophy and for women with the high risk of chromosomal aneuploidies in the embryos [1,2].

Large body of literature documented the use of PGD for high penetrance recessive, dominant and X-liked disorders occurring in early life. It was used as well in case of high penetrance cancer syndromes that appear later [3,4].

Hereditary breast and ovarian cancer (HBOC) reveals as a monogenic predisposition of offspring features autosomal dominant inheritance due to constitutional mutations in the BRCA1 gene [5]. Many BRCA1 gene mutations characterized familial occurrence and the presence of specific mutations are much more frequent in certain isolated populations and ethnic groups compared to the general population [6]. Rubin et al. reported that carriers of mutations in the BRCA1 gene appear to have a significantly more favorable clinical course [7]. In opposite, Johannsson et al. suggested that the survival for carriers of BRCA1 mutation is similar or worse compared to the patients with breast and ovarian cancer in general [8]. Later reports documented that women with BRCA gene mutations have 65–85% risk of breast cancer exposure [9]. The carriers of the BRCA1 and BRCA2 mutation have a risk of the ovarian cancer ranged 18% to 56% and 14% to 27%, respectively [10].

The debate regarding the use of PGD in lower penetrance and late-onset cancer susceptibility syndromes was accomplished with the final UK Human Fertilization and Embryology Authority (HFEA) approval for this diagnostic method to be available for HBOC [11].

Recently published study reported a potential use of PGD for BRCA1/2 carriers, particularly in those who would have to undergo the in-vitro fertilization (IVF) due to the infertility [12]. However these data should be interpreted with caution taking into account the age, emotional stress, fertility status and the presence of confirmed cancer diagnosis [12].

Nowadays, the data regarding the efficacy of different molecular techniques used in PGD are lacking. Nested poly-chain reaction technique (Nested-PCR) and whole genome amplification (WGA) technique remain currently the frequently used methods for genetic mutations in PGD. However their accuracy still remains intriguing due to lacking data regarding the optimal PGD methodology for detecting BRCA1 gene mutations.

Herein we sought to assess the different molecular techniques use in PGD for detecting three most frequent BRCA1 gene mutations: 5382insC, 185delAG and C61G.

## Methods

### Setting

Anonymous donors of the oocytes were extracted in the Fertility and Reproductive Center Invicta in Gdansk. Preimplantation genetic diagnosis for the most frequent BRCA 1 mutations: 185delAG, 5382insC, C61G, was carried out on single, unfertilized oocytes, in metaphase of second meiotic division, not qualified to IVF.

Control blood samples were obtained from the healthy subjects, to perform molecular analysis based on standard procedures carried out in the laboratory of molecular biology, Medical Clinics and Laboratories Invicta, Gdansk, Poland. Positive mutation controls were represented by cell lines from the Coriell Institute for Medical Research: GM14090 (185delAG), GM14097 (C61G), GM13715 (5382insC). The study was approved by the local Ethics Committee in Olsztyn. All patients provided written informed consent before the procedure.

### DNA isolation from lymphocytes and cell lines

The isolation of the genetic material from lymphocytes and cell lines was obtained using a commercial DNA extraction kits (Blood Mini, A&A Biotechnology Comp. and Genomic Mini, A&A Biotechnology Comp., respectively). The measurement of the concentrations of DNA was carried out with a spectrophotometer GeneQuant RNA/DNA Calculator (model 80-2103-98, Pharmacia Biotech, USA) and quartz cuvette, previously washed with distilled water and dried. Each sample was transferred to a cuvette and placed in a chamber of the spectrophotometer. The average results were calculated as appropriate.

### Cell lysis protocol

The cells were deposited in lysis buffer (SDS and proteinase K). Cell lysis was performed by using thermocycler PTC 225 DNA Engine Tetrad Peltier Thermal Cycler according to the profile temperature: 60 min in 37°C, 15 min in 99°C.

### PCR amplification of specific alleles

After cell lysis protocol, to the isolated DNA the outer PCR mix was added. The PCR reaction mix comprises 25 μl of the lysis buffer containing the isolated DNA or negative control, the distilled water. For the mutations 5382insC and C61G the outer PCR reaction contained: 3 μl DNA, 2 μl primers mix, 0,5 μl dNTPs, 0,2 μl polymerase Taq, 2,5 μl PCR buffer, 0,75 μl MgCl_2_ and 16,05 μl H_2_O. For the 185delAG mutation the reaction mix contained: 3 μl DNA, 1 μl primers mix, 0,5 μl dNTPs, 0,2 μl polymerase Taq, 2,5 μl PCR buffer, 0,75 μl MgCl_2_ and 17,05 μl H_2_O. All primers sequences were chosen according to the tested mutation. The sequences of the primers used in the ASA-PCR reaction are presented in Table 1. Amplification for the mutation 5382insC was as follows: denaturation step of 95°C for 120 seconds, followed by 34 cycles of denaturation at 94°C for 60 seconds, annealing at 69°C for 45 seconds, extension at 70°C for 60 seconds and final extension at 72°C for 300 seconds. For the mutations 5382insC, C61G amplification was as follows: denaturation step of 95°C for 120 seconds, followed by 9 cycles of denaturation at 94°C for 60 seconds, annealing at 57°C for 60 seconds, extension at 72°C for 60 seconds. This was followed by 24 more cycles of denaturation at 94°C for 30 seconds, annealing at 57°C for 60 seconds, extension at 72°C for 60 seconds. Final extension was performed at 72°C for 300 seconds.

**Table 1:** The sequences of the primers used in the ASA-PCR reaction

**Mutation**	**Sequence of the primers**	**mp**	**Product length**
5382insC	N: 5’ AGAGAATCCCAGGACA 3’	68,2°C	168 pz
	M: 5’ AGAGAACTCCCAGGAC 3’	81,8°C	
	R: 5’ ATATGACGTGTCTGCTCCAC 3’	64,1°C	
185delAG	N: 5’ GCTGACTTACCAGATGGGACTCTC 3’	66,1°C	335 pz
	M: 5’ CCCAAATTAATACACTCTTGTCGTGACTTACCAGATGGGACAGTA 3’	78,4°C	
	R: 5’ GGT TGG CAG CAA TAT GTG AA 3’	63,4°C	

### Restriction fragments length polymorphism (RFLP)

RFLP was performed to verify the ASA-PCR method. RFLP was carried out for the mutations of C61G. The isolated DNA was fragmented by a restriction enzyme Ava II (EcoR471). Total volume of the PCR reaction mixture was 25 μl and contained: 1,5 μl PCR buffer, 8 μl amplified DNA and 1,5 μl of restriction enzyme. Each PCR reaction contained negative control, the distilled water. Digestion of the restriction enzyme was incubated in 37°C for 12 hours with using Hybrigene incubator (Techne Comp). The sequences of the primers used in the RFLP reaction are presented in Table 2.

**Table 2:** The sequences of the primers used in the RFLP reaction

**Mutation**	**Sequence of the primers**	**mp**	**Product length**
C61G	F: 5’ CTC TTA AGG GCA GTT GTG AG 3’	59,1°C	158 pz
	R: 5’ ATG GTT TTA TAG GAA CGC TAT G 3’	58,6°C	118 pz

### Whole genome amplification (WGA)

We amplified the entire genome from a cell up to microgram level to further analysis. The commercially available Whole Genome Amplification kit (Roche, Switzerland) was used for this study. 1 μl of genomic DNA were mixed with 9 μl sample buffer and then heat denatured at 95°C for three minutes and cooled to 4°C. Next, 9 μl of reaction buffer were mixed with 1 μl enzyme mixture and added to the denatured genomic DNA. The reaction was subsequently incubated at 30°C for 90 minutes, then heat inactivated at 65°C for 10 minutes and cooled on ice. The PCR reaction contained 12,5 μl of the lysis buffer containing the isolated DNA or negative control, the distilled water. Amplification conditions were set for the 5382insC mutation. For the other mutations we did not achieve the expected results.

For the mutation 5382insC the PCR mix reaction contained: 2 μl DNA, 1 μl primers mix, 0,25 μl dNTPs, 0,1 μl polymerase Taq, 1,25 μl PCR buffer, 0,375 μl MgCl_2_ and 8,025 μl H_2_O. All primers sequences were chosen according to the tested mutation. The sequences of the primers used in the ASA-PCR reaction are presented in Table 1. Amplification for the mutation 5382insC was as follows: denaturation step of 96°C for 120 seconds, followed by 10 cycles of denaturation at 96°C for 45 seconds, annealing at 66°C for 45 seconds, extension at 72°C for 45 seconds. This was followed by 20 more cycles of denaturation at 94°C for 45 seconds, annealing at 66°C for 45 seconds, extension at 72°C for 45 seconds. Final extension was performed at 72°C for 300 seconds.

### Nested-PCR amplification

Primers sequences used in the nested-PCR reaction were designed based on the published sequences of the BRCA1 gene in a ESEMBL ( http://www.ensembl.org; BRCA1: ENST00000337272) and using the Primer3 program for primer design.

Nested PCR reaction is based on the amplification of an extended sequence in the first stage and the amplification of an internal sequence from the product of the first in second stage. This technique increases specificity and sensitivity of the PCR reaction by controlling reaction conditions for each amplification to favor generation of the desired product.

#### Nested-PCR reaction for blood samples

Multiplex-PCR reaction was carried out for the mutations of BRCA 1 gene: 5382insC, 185delAG and C61G. DNA was obtained from unrelated individuals of Polish population and isolated from peripheral blood lymphocytes. Total volume of the PCR reaction mixture was 25 μl and contained: 0,5 μl of the isolated DNA, 3 μl primers mix, 0,5 μl dNTPs, 0,2 μl polymerase Taq, 2,5 μl PCR buffer, 0,75 μl MgCl_2_ and 17,55 μl H_2_O. Each PCR reaction contained negative control, the distilled water. The sequences of the primers used in the nested-PCR reaction are presented in Table 3. First stage of amplification for the mutations 5382insC, 185delAG and C61G was as follows: denaturation step of 95°C for 120 seconds, followed by 34 cycles of denaturation at 94°C for 60 seconds, annealing at 62°C for 60 seconds, extension at 72°C for 60 seconds and final extension at 72°C for 300 seconds. In second stage of amplification the PCR reaction mixture for the mutation 5382insC contained: 0,5 μl of the isolated DNA, 2 μl primers mix, 0,5 μl dNTPs, 0,2 μl polymerase Taq, 2,5 μl PCR buffer, 1,12 μl MgCl_2_ and 18,18 μl H_2_O. The annealing temperature was raised from 69°C to 71°C. The PCR reaction mixture for the mutation 185delAG contained: 0,5 μl of the isolated DNA, 1 μl primers mix, 0,5 μl dNTPs, 0,2 μl polymerase Taq, 2,5 μl PCR buffer, 0,75 μl MgCl_2_ and 19,55 μl H_2_O. The annealing temperature was raised from 57°C to 65°C. Each PCR reaction contained negative control, the distilled water.

**Table 3:** The sequences of the primers used in the nested-PCR reaction

**Mutation**	**Sequence of the primers**	**mp**	**Product length**
5382insC	F: 5’ AGTGATCTGCCTGCCTCAGT 3’	64,1°C	315 pz
	R: 5’ CCATCTCTGCAAAGGGGAGT 3’	65,4°C	
185delAG	F: 5’ TTGGAGAAAGCTAAGGCTACCA 3’	65,4°C	685 pz
	R: 5’ CCCAGTGCAGAACCAATCA 3’	64,9°C	
C61G	F: 5’ TTGCTTATGCAGCATCCAAA 3’	64,2°C	633 pz
	R: 5’ GCACTCCAGCCTCAGTGAC 3’	63,9°C	

**Table 4:** WGA of oocytes-recurrence of the results for oocytes obtained from the same amplification conditions for each mutation

**Mutation**	**Amount of amplified gene fragments in one sample**	**Number of samples [n]**	**Average**	**Recurrence**
5382insC	3/3	2	0,38	38%
	0/5			

#### Nested-PCR reaction for oocytes

DNA was isolated from single oocytes. Multiplex-PCR was performed for each mutations of BRCA1 gene: 5382insC, 185delAG and C61G. The PCR reaction was performed in a total volume of 25 μl with negative control–the distilled water. The sequences of the primers used in the nested-PCR reaction are presented in Table 3. The composition of the PCR reaction mixture was the same as in the second stage of amplification for blood samples. The amount of isolated DNA was increased to 6 μl in the second stage of amplification. The PCR reaction was performed in a total volume of 25 μl and for mutation of 5382insC contained: 6 μl of DNA, 2 μl primers mix, 0,5 μl dNTPs, 0,2 μl polymerase Taq, 2,5 μl PCR buffer, 0,75 μl MgCl_2_ and 13,05 μl H_2_O. For the mutations of 185delAG and C61G PCR reaction contained: 6 μl of DNA, 1 μl primers mix, 0,5 μl dNTPs, 0,2 μl polymerase Taq, 2,5 μl PCR buffer, 0,75 μl MgCl_2_ and 14,05 μl H_2_O. In the third stage of amplification were performed ASA-PCR and RFLP analyses. PCR products were separated on a 2% denaturing polyacrylamide gel mounted on a Sigma-Aldrich DNA Sequencer and Power-Pac 300 (Bio-Rad) with automated fluorescent scanning detection and analyzed using Quantity One Analysis Software (Bio-Rad).

## Results

**Figure 1:**
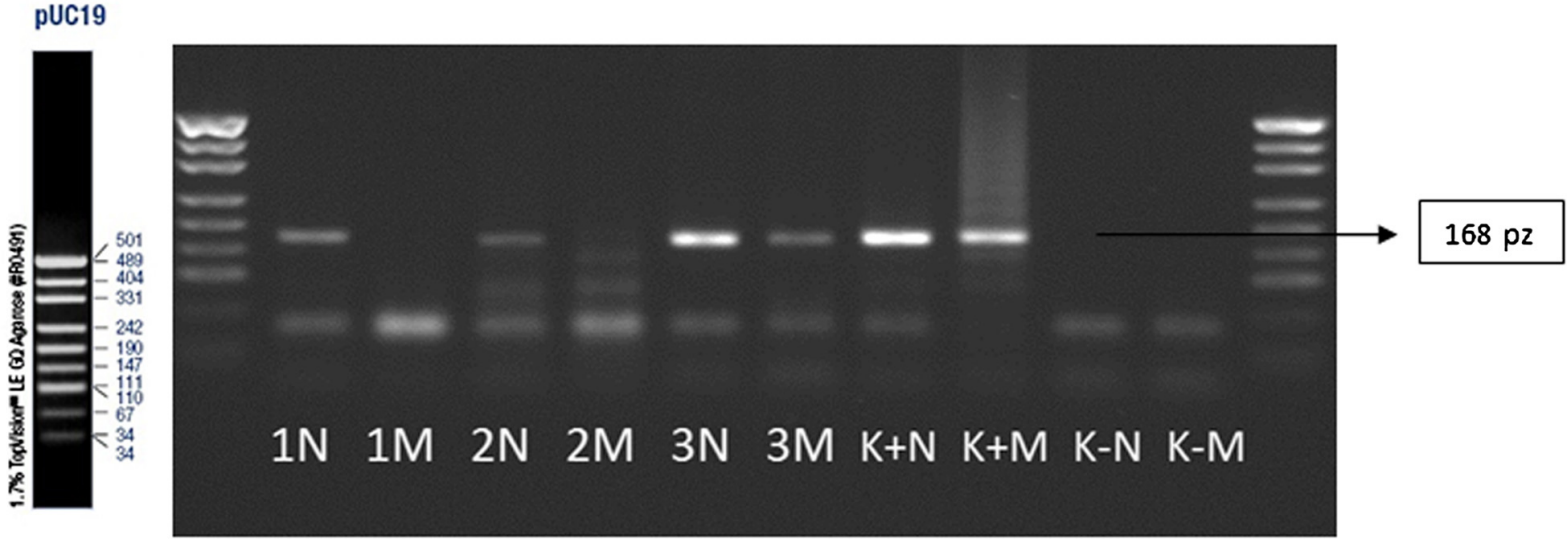
**WGA analysis-ASA-PCR technique.** Analyses of the mutation of 5382insC; 1 N, 2 N, 3 N, 4 N–normal allele; 1 M, 2 M, 3 M, 4 M–mutation allele; K+N, K+M–positive control (N–normal alelle, M–mutation allele); K-N, K-M–negative control; size marker–pUC19.

**Figure 2:**
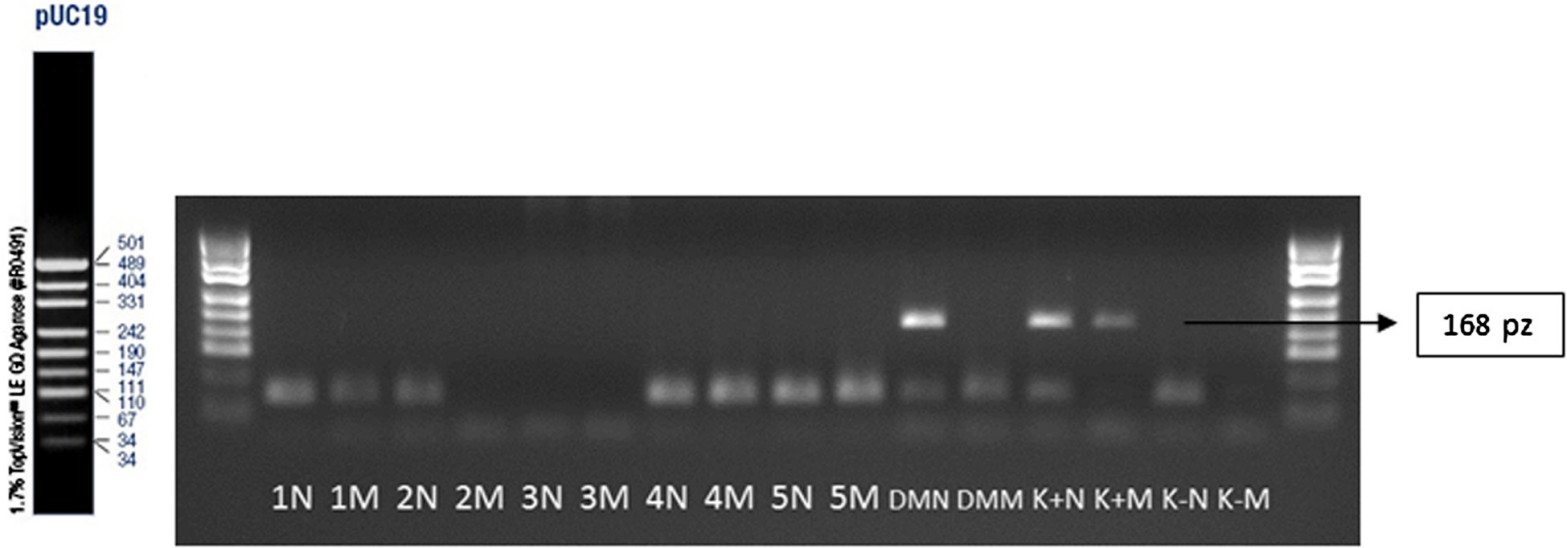
**WGA analysis-ASA-PCR technique.** Analyses of the mutation of 5382insC; 1 N, 2 N, 3 N, 4 N–normal allele; 1 M, 2 M, 3 M, 4 M–mutation allele; K+N, K+M–positive control (N–normal alelle, M–mutation allele); K-N, K-M–negative control; size marker–pUC19.

**Figure 3:**
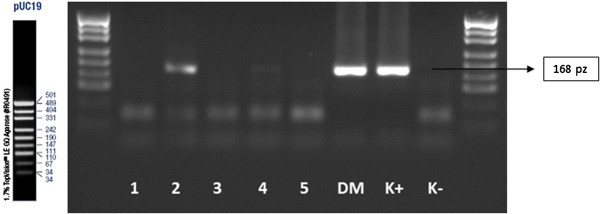
Nested-PCR amplification of oocytes, IInd amplification–analysis of the mutations of 5382insC; 1, 2, 3, 4, 5–amplificated fragment of the gene; DM–amplificated fragment of the gene on blood sample probe; K+ –positive control; K- –negative control; size marker–pUC19.

**Figure 4:**
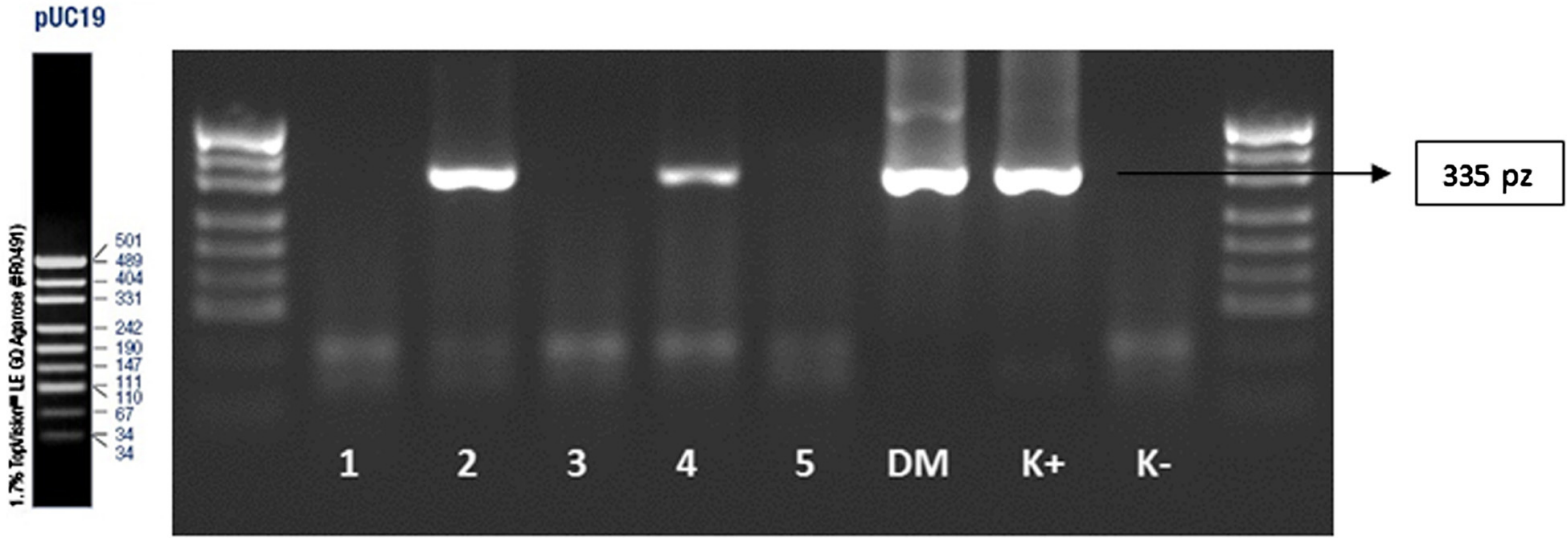
Nested-PCR amplification of oocytes, IInd amplification–analysis of the mutations of 185delAG; 1, 2, 3, 4, 5–amplificated fragment of the gene; DM–amplificated fragment of the gene on blood sample probe; K+ –positive control; K- –negative control; size marker–pUC19.

**Figure 5:**
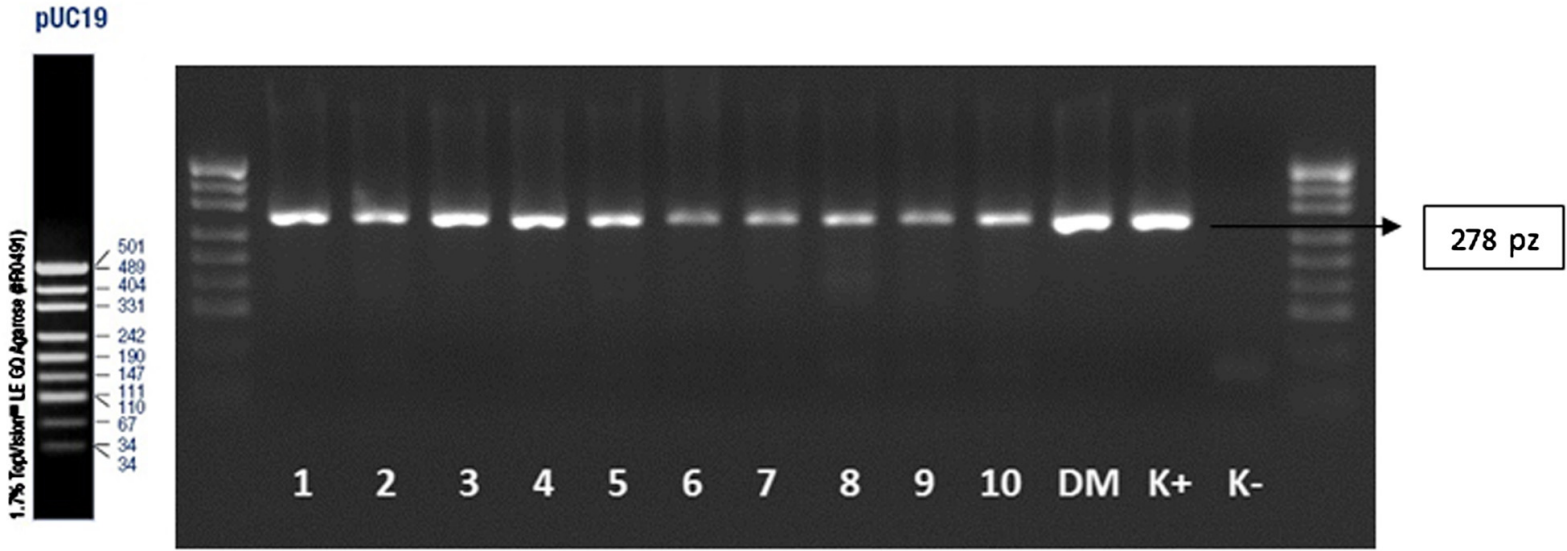
Nested-PCR, IInd amplification–analysis of the mutations of C61G; 1, 2, 3, 4, 5, 6, 7, 8, 9, 10-amplificated fragment of the gene; DM–amplificated fragment of the gene on blood sample probe; K+ –positive control; K- –negative control; size marker–pUC19.

**Figure 6:**
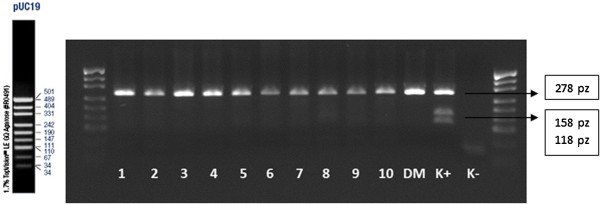
Nested-PCR–analysis of the mutations of C61G after enzyme restriction Ava II in 37°C temp.; 1, 2, 3, 4, 5, 6, 7, 8, 9, 10-amplificated fragment of the gene; DM–amplificated fragment of the gene on blood sample probe; K+ –positive control; K- –negative control; size marker–pUC19.

Repeatability of the results acquired from the WGA analysis for the mutation 5382insC was 38% (Table 4). The WGA analysis for the mutation 5382insC of BRCA 1 gene is presented on Figures 1 and 2. WGA performed on the single cells did not reflect expected results. The repeatability of this method remains still questionable particularly for the material investigated in the present study. The repeatability of the nested-PCR analysis in the second round of the amplification was labile for the mutation 5382insC and 185delAG and was ranged from 47% to 57% (Table 5). However, the repeatability for the mutation C61G was 100% (Table 5). Sample results of the nested-PCR analysis for each mutation of BRCA 1 gene are presented on Figures 3, 4, 5 and 6.

**Table 5:** Nested-PCR amplification of oocytes–second stage of amplification-recurrence of the results for oocytes obtained from the same amplification conditions for each mutation

**Mutation**	**Amount of amplified gene fragments in one sample**	**Number of samples [n]**	**Average**	**Recurrence**
5382insC	4/5	3	0,47	47%
	2/5			
	1/5			
185delAG	2/5	3	0,67	67%
	3/5			
	5/5			
C61G	5/5	2	2	100%
	10/10		1	

## Discussion

The present study reports a comparison of two PGD techniques: WGA and nested-PCR. Our results suggest that the nested-PCR technique is more sensitive and specific method as compared to WGA potentially due to the two-phase amplification which simplifies the final analysis. The usage of the WGA method therefore remains questionable, and any analysis attempt does not guarantee reliable results.

Among female population, 5000 breast cancer deaths is recorded in Poland annually [13]. Morbidity rate rises with the age and is the highest at 40 years of age. The genetic basis of cancer significantly worses patients clinical status.

Breast cancer susceptibility gene 1 (BRCA1) was the first gene assigned responsible for developing hereditary breast and ovarian cancer which manifests as a HBOC syndrome (Hereditary Breast and Ovarian Cancer Syndrome). HBOCs reveals as a monogenic predisposition of pedigree features autosomal dominant inheritance due to constitutional mutations in the BRCA1 gene. According to the dominant model of inheritance of just one allele mutation that predisposes to disease BRCA1 mutated gene is passed to offspring by both men and women. The BRCA1 gene has almost 2000 described sequences changes, including mutations leading to changes in the reading frame by deletion or insertion, missense mutations, inframe deletions, nonsense and various types of sequence variants and polymorphisms (Breast Cancer Information Core). Most of them lead to the premature termination of translation and a shortened protein formation (approximately 87% of all terminal mutations in BRCA1). It has been suggested that the clinical effect depends on the position along the gene mutation. The central region of the BRCA1 gene is associated with an increased risk of breast cancer compared to other regions of the gene. Furthermore, the mutations are located closer to the 5 ′end of the BRCA1 gene are at higher risk for ovarian cancer than those to the 3′ end [14,15].

However a number of studies were performed among Polish population, data regarding the methodology of gene mutations detection are lacking [16-21]. The preimplantation diagnosis technique has not been published so far. Therefore, this paper presents a methodology for detection of three mutations of BRCA 1 gene: 5382insC, 185delAG and C61G, using unfertilized oocytes from anonymous women. Here we should depict the fact, that the mutations in the BRCA1 gene do not constitute a pathogenic factor, but potentially predispose to breast cancer.

Preimplantation genetic diagnosis (PGD) is nowadays a valid alternative for parents with a genetic disease and high-risk child transmission of gene defects. This remains also an alternative for women older than 37–40 years with the high risk of chromosomal aneuploidies in the embryos [1,2]. The usage PGD for lower penetrance and late-onset cancer susceptibility syndromes is still debated and remains a frequently asked and as yet incompletely answered question.

In the current study we performed two most common PGD techniques of molecular biology-WGA and nested multiplex PCR. It was demonstrated that none of these diagnosis methods of mutations in the BRCA1 gene were satisfactory due to its low effectiveness.

The main disadvantages of WGA technique is non-specific products formation. In the multiplex nested-PCR technique the most challenging is appropriate primers design for the multiplex reaction. Although this method is more specific with respect to the studied gene segment. Moreover, the use of several pairs of primers allows receiving a proper short DNA fragment.

However, the multiplication of genetic material during subsequent rounds of amplification can also lead to the formation of nonspecific products. Proper analysis depends on the appropriate diagnostic technique. Therefore we obtained positive results for diagnostic analysis of the C61G mutation using RFLP technique. Recurrence of nested-PCR method for single oocyte in the second round of amplification was 100%. ASA-PCR technique has proven to be sub-optimal diagnostic method. After using the WGA technique recurrence was 38%. In the nested-PCR technique recurrence was 47% for mutation 5382insC and 67% for 185delAG mutation.

In 2007, Menon and colleagues published the results of a postal survey of BRCA mutation carriers. Only 51% of patients responded to the previously send questionnaires. Of these, 75% were supportive of offering PGD [11]. 15 people from 40 respondents (37.5%) who do not plan to enlarge the family and 14% of those who want to enlarge the family would consider the PGD [11]. Another study performed by Quinn et al. presented that out of 111 patients with strong aggregation of breast and ovarian cancer, 57% found PGD as a one of the diagnostic methods of BRCA1 mutation in carriers, while the other 33% were personally interested in using this method [22].

Sagi et al. published a method for PGD, where convinced that the majority of patients participating in the program were often very pleased with the opportunities given by PGD [12]. It should be noted that the study performed on patients with breast and ovarian cancer HBOCs has a relatively high level of acceptance of PGD for the BRCA1 gene mutation diagnosis. Interest in PGD procedure is ranged between 14 and 75%.

Undoubtedly, PGD in mutations of the gene BRCA1 will be often accepted by patients where in vitro fertilization is necessary. However, it should be emphasized, that fertile patients would also consider the possibility of performing PGD techniques, particularly in case of the strong aggregation of breast cancer within the family with the occurrence of cancer at a young age. In addition in families of patients with co-occurrence of breast and ovarian cancer, significantly increases the probability of identification of the mutation in this gene. Certainly, the decision is impacted by the ethical and psychological factors.

Moreover, we should stress the fact that PGD for the mutation of BRCA1 gene is still controversial and meets with multiple opponents. However, PGD should be considered for people who have fertility problems and are burdened to genetic mutation in the BRCA1 gene. Nonetheless, mutations in the BRCA1 gene do not give confidence to transform normal cells into cancer cells, and other factors are essential to conduct neoplasia. PGD is therefore addressed to carriers the mutation in the BRCA1 gene wanted to deprive their future children a substantial risk of breast cancer.

It seems that the lack of knowledge of patients about the hereditary breast and ovarian cancer, can significantly reduce the level of trust in relation to preimplantation diagnosis.

Although PGD for the BRCA1 gene mutation detection remains still controversial, there is a large group of people who would decide to perform the PGD. The knowledge of the potential consequences of the gene mutation possession will raise the desire to explore opportunities carried by PGD. The awareness of having the mutated gene and the ability of transferring gene defect to offspring should significantly sensitize the patient to the future fate of their children. Unfortunately, most people still do not have sufficient knowledge of PGD or never heard of this diagnostic technique, which significantly affects the current opinions. The accurate methodology of PGD would also strengthen the efforts to broad the knowledge of the positive sites of PGD and improve its use in routine clinical practice.

## Conclusion

In the study we compared the two PGD techniques: WGA and nested-PCR. Our results suggest that the nested-PCR technique is more sensitive and specific method as compared to WGA potentially due to the two-phase amplification which simplifies the final analysis. WGA performed on the single cells did not reflect expected results. The repeatability of the WGA methodology remains questionable, and any analysis attempt does not guarantee reliable results.

The accuracy of different PGD methodologies for detecting BRCA1 gene mutations still remains intriguing due to the lacking data so far. Therefore further evaluation is strongly needed to propose the most accurate molecular technique used in PGD for detecting three most frequent BRCA1 gene mutations: 5382insC, 185delAG and C61G.

## Competing interests

The authors report no financial relationships or conflicts of interest regarding the content herein.

## Authors’ contributions

KL - study concept and design. DM, JL, KK, AM - carried out the molecular genetic studies and participated in the sequence alignment. DM, JL, KJ, KL - interpretation of data. KJ - drafted the manuscript. KL - critical revision of the manuscript for important intellectual content. KL - study supervision. All authors read and approved the final manuscript.
